# Performance of Linear Mixed Models to Assess the Effect of Sustained Loading and Variable Temperature on Concrete Beams Strengthened with NSM-FRP

**DOI:** 10.3390/s21155046

**Published:** 2021-07-26

**Authors:** Ricardo Perera, Lluis Torres, Francisco J. Díaz, Cristina Barris, Marta Baena

**Affiliations:** 1 Department of Mechanical Engineering, Technical University of Madrid, 28006 Madrid, Spain; franciscojavier.diaz.nunez@alumnos.upm.es; 2 Analysis and Advanced Materials for Structural Design (AMADE), Polytechnic School, University of Girona, 17003 Girona, Spain; lluis.torres@udg.edu (L.T.); cristina.barris@udg.edu (C.B.); marta.baena@udg.edu (M.B.)

**Keywords:** NSM-FRP strengthening, mixed effects model, structural health monitoring, PZT sensors, electro-mechanical impedance, sustained load, temperature

## Abstract

Although some extended studies about the short-term behavior of NSM FRP strengthened beams have been carried out, there is a lack of knowledge about the behavior of this kind of strengthening under sustained loads and high service temperatures. Electromechanical impedance method formulated from measurements obtained from PZT patches gives the ability for monitoring the performance and changes experienced by these strengthened beams at a local level, which is a key aspect considering its possible premature debonding failure modes. This paper presents an experimental testing program aimed at investigating the long-term performance of a concrete beam strengthened with a NSM CFRP laminate. Long term performance under different levels of sustained loading and temperature conditions is correlated with EMI signatures processed using Linear Mixed-effects models. These models are very powerful to process datasets that have a multilevel or hierarchical structure as those yielded by our tests. Results have demonstrated the potential of these techniques as health monitoring methodology under different conditions in an especially complex problem such as NSM-FRP strengthened concrete structures.

## 1. Introduction

Strengthening of reinforced concrete (RC) beams using near surface mounted (NSM) fiber-reinforced polymer (FRP) strips and bars has gained greater interest and increased field applications in recent years. In comparison with the more extended methodology of FRP strengthening based on externally bonded reinforcement (EBR), NSM FRP technique presents several advantages such as better anchorage capacity and better protection from accidental damage as those due to, for instance, vandalism and environmental effects [1,2,3]. Additionally, NSM FRP strengthening does not need much surface preparation except for grooving. However, similarly to EBR FRP, premature debonding failure becomes a critical point when designing NSM FRP strengthened RC structures. Therefore, the damage needs to be detected in advance or in real time to ensure structural safety and to predict the residual life of FRP composite RC structures in service.

Extensive studies about the short-term behaviour of NSM FRP strengthened beams have been carried out [4,5]. However, little attention has been paid to its performance under sustained load despite this action might affect its mechanical performance [6,7]. The same limitations exist under high temperature conditions [8,9]. It is evident that there is a lack of knowledge on the performance of this kind of strengthening and the experienced changes under sustained loads and high temperatures and, therefore, more research work needs to be developed.

The application of smart piezoelectric transducers (PZT) for the structural health monitoring (SHM) of FRP composite RC structures is a relatively innovative method since some studies have been already developed in this area [10,11,12,13,14]. However, in the case of NSM FRP technique, the number of studies is practically non-existent [15].

There are mainly two main detection approaches based on the use of PZTs, wave propagation and electromechanical impedance (EMI). In wave propagation [16], the structure is excited by one PZT which generates a stress wave. After propagating along the inspected structure, the wave is captured by other PZT where is converted into an electrical signal. By analyzing the signal, a diagnostic of the structure can be made. In the EMI technique [17], the same PZT is used as actuator and sensor in such a way that the structure is excited with the sensor using the converse piezoelectric effect and the mechanical response is captured by the same PZT using the direct piezoelectric effect. The electrical impedance, measured by an impedance analyzer, serves to evaluate the damage.

This paper aims to contribute to the study of the behavior of RC beams strengthened with NSM FRP strips under the combined action of sustained loading and temperature variations which, to the knowledge of the author, has not been carried out up to date. For it, an experimental testing program aimed at investigating the long-term performance of a concrete beam strengthened with a NSM CFRP laminate is performed. The program has been implemented specifically to address the performance of NSM FRP strengthening systems under different sustained load levels and at elevated temperatures. EMI method will be used for monitoring the performance and changes of this strengthened beam. The EMI signatures are recorded using FRP reinforcement and concrete surface bonded PZT sensors. However, the correlation of the long term performance under different levels of sustained loading and temperature conditions with EMI signatures is not easy, considering the complexity of the captured experimental data and the coupling between mechanical and temperature effects in the impedance measurements. A suitable analysis tool should be used to get a successful interpretation of the data. Linear Mixed-effects Models (LMMs) [18,19] as a tool defined from different statistic metrics such as root mean square deviation (RMSD) and mean absolute percentage deviation (MAPD) [17] will be used. Linear Mixed-effects Models (LMMs), also known as multilevel or random effects models, are a statistical analysis tool for predicting scenarios and are of extended application in fields such as meteorology, biology, psychological science and medical sciences, etc. [20,21,22]. However, its application in structural monitoring is practically inexistent [23], even although it might become the default approach in the future to analyze quantitative data in this area. Because of it, another important contribution of this work is the evaluation of the performance of this approach when applied to a complex structural health monitoring problem in which mechanical and thermal effects are coupled.

## 2. EMI Method

In the EMI technique, the same PZT is used as actuator and sensor in such a way that the structure is excited with the sensor using the converse piezoelectric effect and the mechanical response is captured by the same PZT using the direct piezoelectric effect. The mechanical impedance of the host structure (Z_s_) and that of PZT sensor (Z_a_) can induce an electrical impedance of PZT, which is the reciprocal of the admittance (Y). EMI technique was firstly developed by Liang et al. [24] who proposed the PZT’s theoretical admittance model as shown below

(1) $$Y\left(\omega \right)=G\left(\omega \right)+\mathrm{jB}\left(\omega \right)=j\omega \frac{\mathrm{wl}}{h}\left({\bar{\varepsilon }}_{33}^{T}-\frac{Z_{s}\left(\omega \right)}{Z_{s}\left(\omega \right)+Z_{a}\left(\omega \right)}d_{3x}^{2}{\hat{Y}}_{\mathrm{xx}}^{E}\right)$$

where the admittance is a function of the conductance (G) and the susceptance (B) with its imaginary unit (j) and depends on the PZT sensor dimensions (width, w, length, l, and height, h), the electrical permittivity (${\bar{\varepsilon }}_{33}^{T}={\;\varepsilon }_{33}^{T}\left(1-\delta j\right)$), a piezoelectric coefficient (d31) and the complex dynamic Young’s modulus ${\hat{Y}}_{\mathrm{xx}}^{E}$; δ is the dielectric loss factor.

By quantifying the variations of the electrical impedance with respect to a baseline signature, a diagnosis of the structural health can be made. For it, three different statistical models, RMSD (root mean square deviation), CCD (correlation coefficient deviation), and MAPD (mean absolute percentage deviation) can be used.

(2) RMSD(%)=∑i=1N[Re(Z1(ωi))−Re(Z0(ωi))]2∑i=1NRe(Z0(ωi))2·100

(3) CCD(%)=100−{1NσZ0σZ1∑i=1N[(Re(Z1(ωi))−Re(Z1¯(ωi)))][(Re(Z0(ωi))−Re(Z0¯(ωi)))]}

(4) MAPD(%)=1N∑i=1N|Re(Z1(ωi))−Re(Z0(ωi))Re(Z0(ωi))|

where $Z_{0}\left(\omega_{i}\right)$ is the baseline impedance spectra, $Z_{1}\left(\omega_{i}\right)$ is the spectra corresponding to the different stages of the structure, and N is the number of frequency data points in the EMI spectra; $\bar{Z}$ and σ represent the mean and the standard deviation, respectively. The real part of the impedance is regularly used because it is more sensitive to the changes experienced by the structure.

## 3. Experimental Programme

### 3.1. Test Set-Up

To characterize the behavior of the NSM-FRP strengthening system under sustained loading and different conditions of temperature, a reinforced concrete specimen strengthened with this system was used. The beam was loaded with a four-point bending test.

The material properties of the concrete, the reinforcement steel, and the FRP were the following: (a) Concrete: f_c_ = 30 MPa, E_c_ = 26 GPa, f_ct_ = 3 MPa; (b) Steel: f_y_ = 500 MPa, E_s_ = 210 GPa; (c) CFRP: f_fu_ = 2500 MPa, E_f_ = 170 GPa.

**Figure 1:**
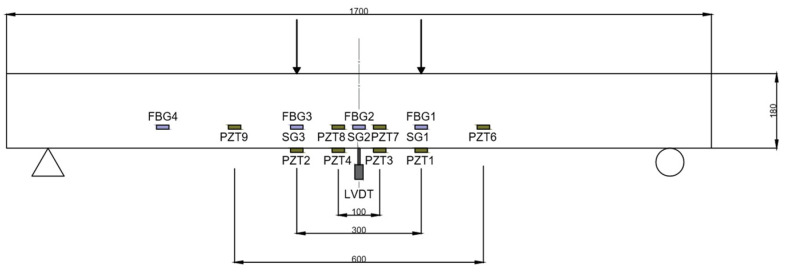
Test set-up.

**Figure 2:**
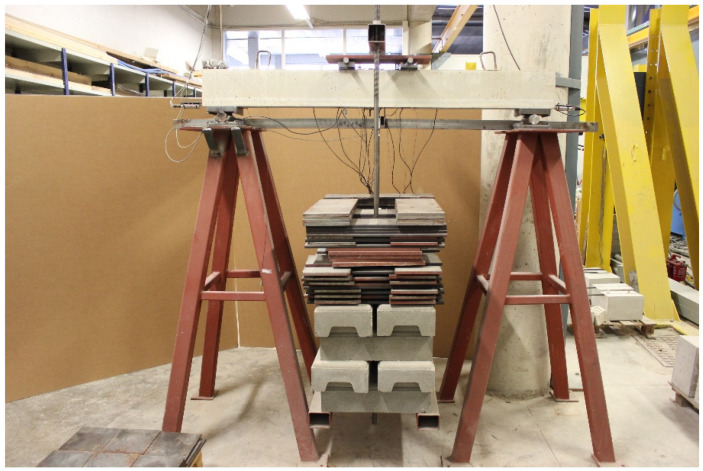
Experimental set-up.

The test set-up and instrumentation are shown in Figure 1 and Figure 2. One LVDT was put in the middle section of the beam to measure its vertical deflection and one electrical resistance strain gage was glued to the center of the compressed concrete surface. Likewise, the beam was instrumented with three FBG sensors, FBG1, FBG2, and FBG3, bonded to the NSM-FRP laminate at both sides of the middle section of the specimen, such as shown in Figure 1. FBG sensors were connected to a Micron Optics sm130-700 Optical Sensing Interrogator and the strain gages were connected to the strain gage data logger. As in the FBG sensors the peak Bragg wavelength shifts proportionally with the variations of axial strain and temperature, these optical sensors can be utilized as a temperature or strain sensing unit. Because of it, a temperature compensation FBG sensor, FBG4 in Figure 1, has been also mounted on the FRP bar. This type of sensor is packaged such that it is isolated from mechanically induced strain when properly mounted on a specimen. In this way, it is ideal for temperature compensation of other optical strain gages.

For the EMI tests, eight PZT patches (PZT1 to PZT2 and PZT5 to PZT9 according to Figure 1) were bonded in different locations of the specimen to be analyzed. Inner sensors were directly bonded to the FRP laminate. PZT1 and PZT2 are sensors of type P-876.A12 (61 mm × 35 mm× 0.5 mm), while sensors PZT3 to PZT9 are sensors of type P-876.SP1 (16 mm × 13 mm × 0.5 mm). All of them are provided by Piceramic [25].

During the EMI tests, the impedance analyzer excites each bonded PZT patch using a voltage of 1 V with a frequency range between 10 kHz and 100 kHz and a frequency step of 12.5 Hz. The response of each PZT’s impedance is recorded with the same Agilent 4294A Impedance Analyzer, which is connected to a computer with a data acquisition software implemented in VEE. Five frequency sweeps were conducted for each sensor at each test, resulting in five impedance signals that were averaged in order to obtain the impedance signal that would be later on used for the analyses.

### 3.2. Loading Procedure

The loading history applied to the specimen typically consisted of several sustained four-point loading tests and their subsequent unloading. Five different levels of increasing sustained loading were applied along the history, 8, 9.3, 13.7, 17.7, and 19.6 kN. The first three levels correspond to the cracking load plus 15%, 35%, and 100%, respectively. The fourth level is associated with the steel reinforcement yielding and, finally, in the fifth level, being the steel reinforcement in the yielding state, the increment of internal tensile force was practically supported by the FRP reinforcement. For each level, the test was repeated several times with different time sequences. Additionally, along the loading history, the beam was also subjected to some temperature increments provided by the heaters. Details of the loading history are shown in Table 1.

In Table 1, the first column contains the number of the test, while in the second column the end date of each test is shown. The applied sustained load level previous to each impedance test is identified in the third column. The fourth and fifth columns show the duration and the temperature, respectively. After each load test, the beam is unloaded previously to the impedance test. Temperature tests are always performed on the unloaded beam and the corresponding electromechanical impedances are measured after one or several days under sustained temperature. Furthermore, a new impedance test is always made once the beam has returned to the environmental temperature.

For each impedance test, five frequency sweeps were conducted for each sensor, resulting in five impedance signals that were averaged to obtain the impedance signal that would be later on used for further analysis.

PZT2 sensor came partially unglued from test 17 and, from that moment, its measurements were not considered in the study.

### 3.3. Results

**Figure 3:**
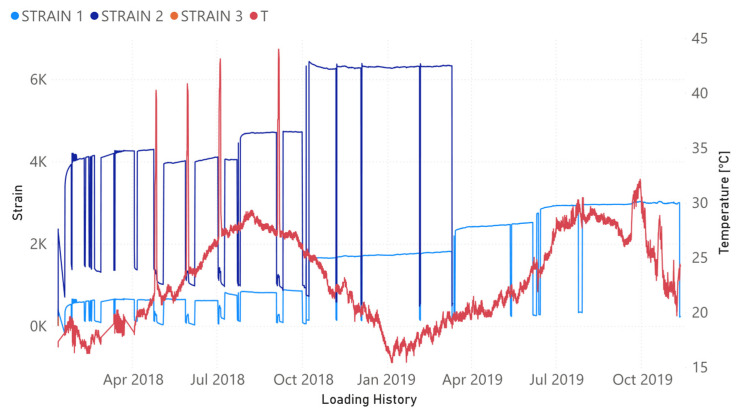
Evolution of strain/temperature along the loading history.

**Figure 4:**
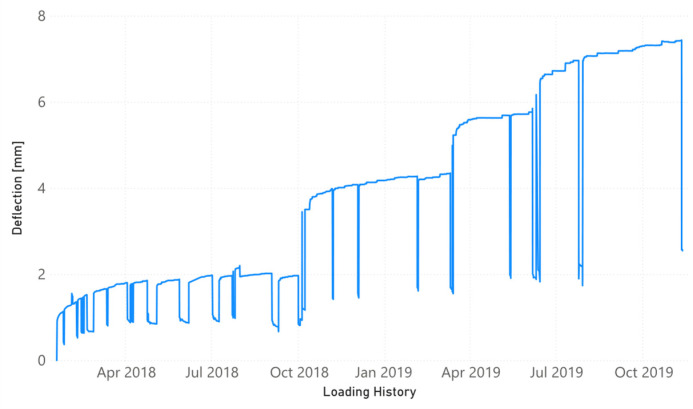
Evolution of midspan deflection along the loading history.

**Figure 5:**
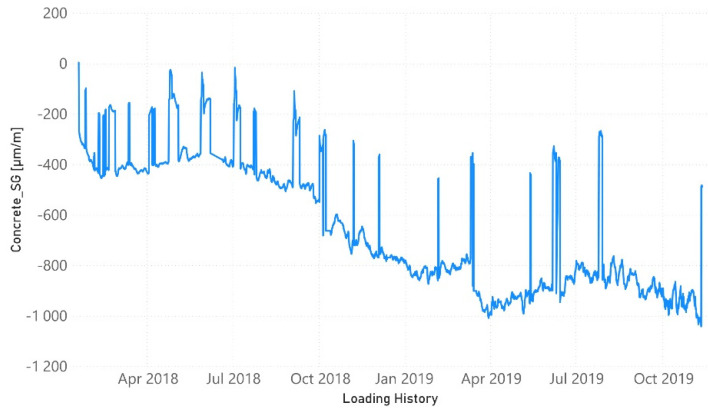
Evolution of compressive strain along the loading history.

Figure 3 presents the evolution of the strain (sensors FBG1, FBG2, and FBG3) and the temperature (FBG4) for the four optical sensors along the loading history. In the same way, the evolution of the vertical midspan deflection as well as the compressive strain in the upper concrete surface are shown in Figure 4 and Figure 5, respectively. From the very beginning, FBG3 sensor did not work correctly and, therefore, the orange line in Figure 3 is only slightly visible initially. Strain values are shown in Figure 3 once temperature compensation has been performed.

Although Figure 3, Figure 4 and Figure 5 show the results until November 2019, the last impedance test was performed at the end of July 2019 (Table 1). The load increments along the time are perfectly reflected by the increment experienced by the strains. However, because of the short duration of the sustained load tests, the creep behavior was little developed. FBG2 sensor, located at the midspan of the beam, did not already work correctly when yielding initiated and its value is only shown until that moment. A remanent strain remained at the midspan of the NSM-FRP laminate after each unloading. One interesting aspect to remark is that this strain is lower whenever the level of sustained load previously reached is higher.

The four levels of artificial heating of the specimen are captured by the FBG4 temperature sensor. In the same way, the environmental temperature variations along the different year seasons are also identified in Figure 3. From September 2019, some instabilities in the measurements of FBG4 sensor demonstrate that, probably, this sensor was already partially unglued.

## 4. LMM Analysis

### 4.1. Linear Mixed Model

Linear mixed models involve a generalization of linear regression but with both fixed and random effects and could represent an appropriate statistical procedure to deal with intersensors variability. Both fixed and random effects in linear mixed-effects models occur linearly in the model function. These models are particularly used when there is not independence in the data, such as arises from a hierarchical structure as the tests performed in this work are. The data set-up of these tests corresponds to repeated measures or multistage sampling where correlations among the experiments are likely.

The study of the changes experienced by a specimen generally involves a repeated monitoring of each of the sensors installed on the specimen. Two sources of variability might appear in the repeated datasets: the variability between the observations measured on the same sensor along the time and the variability between the sensors themselves. The mixed model is a statistical tool allowing highlighting the relationship between the observed response and explanatory covariates, considering these two types of variations.

In this case, the observed response consists in the impedance measurements captured from different PZT transducers bonded to the beam. This response has been condensed by RMSD coefficient. The Linear mixed model is used to model the relationship between RMSD index and the variations of the beam by considering each sensor individual response as a random effect. These variations will affect the value of RMSD index and are referred to as fixed effects. Additionally, if the variations are due to mechanical damage, the model would be useful as indicator about the real condition of the structure, although other effects, such as thermal changes, will also originate variations. The mixture of both fixed and random effects is what gives name to the mixed model.

The use of MAPD metrics has been also evaluated, but the conclusions are similar to RMSD; because of it, only the results obtained with RMSD will be presented.

Formally, the assumptions of a linear mixed-effects model involve validity of the model, independence of the measurements from each other, linearity of the relationship between the predictor and response, homogeneity of the residuals or homocedasticity and normality of the residuals.

### 4.2. Preliminary Analysis

**Figure 6:**
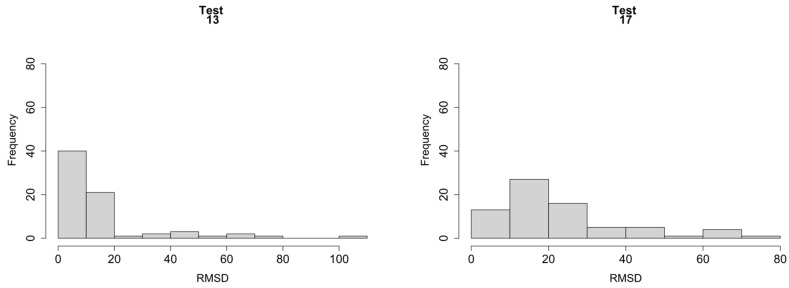
RMSD histograms.

Initially, before performing the statistical analysis, means and standard deviations were calculated for the variable RMSD. As a sample, Figure 6 shows the RMSD histograms for tests 13 and 17 according to Table 1. Test 2 was taken as the reference stage to compute RMSD values since previous to this stage the specimen was moved which required the disconnection and subsequent connection of the measurement equipment. From Test 2, the beam remained permanently installed in the same location and the equipments were not disconnected again. It is clear that RMSD was not normally distributed for tests 13 and 17. Although not shown her by simplicity, the conclusion would be the same for the other tests.

Normal distribution of the residuals (difference between the observed values and the model-estimated values) was also verified by the Shapiro–Wilk test when all results from the experimental tests were combined. A *p*-value (probability of our data following a normal distribution given the dataset) lower than 2 × 10^−16^ was computed, which indicates a significant departure from the normal distribution in the residuals of the model.

**Figure 7:**
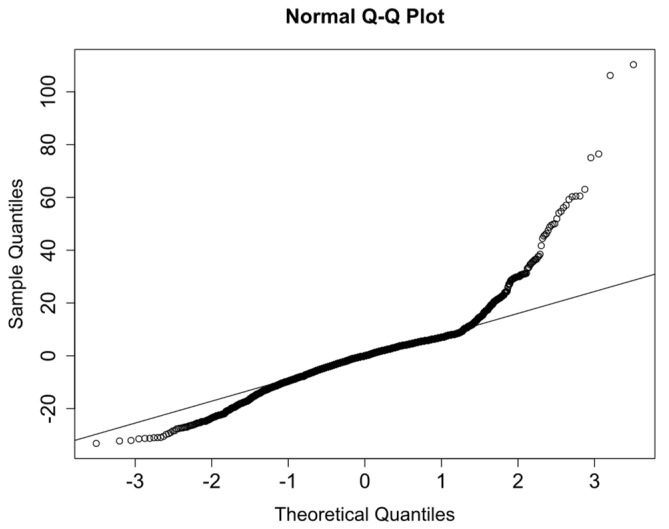
Q-Q plot.

Non-normality is also confirmed by the quantile-quantile plot (Q-Q plot) (Figure 7). The strong deviation from the provided line is a clear symptom of non-normality of the residuals.

**Figure 8:**
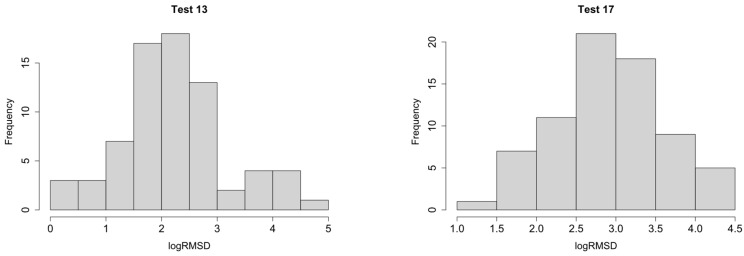
logRMSD histograms.

Because of the non-normality of the residuals, we will work with a log-transform of the response to check if this condition is improved. Kolmogorov-Smirnov test will allow to know if the data have a significant departure from log-normality. Table 2 shows the *p*-values for all tests considering all sensors. The interpretation of *p*-value is similar to the Shapiro-Wilk test. Results demonstrate that, except for test 32, our data follow a log-normal distribution and, therefore, the LMM analysis will be made using logRMSD. As a sample and for comparison with Figure 6, Figure 8 shows the logRMSD histograms for tests 13 and 17.

As previously commented, we can perform analyses across all measurements. However, results from the analysis could vary between sensors since different types of sensors and different locations (embedded or external surface bonded) were used in the test. Therefore, the intersensor variability should be taken into account when analyzing the data to avoid inaccurate results. For the analysis, four groups of sensors were considered according to Figure 1. Group 1 includes sensors PZT1 and PZT2, which are the larger surface-bonded sensors. Group 2 includes sensors PZT3 and PZT4, which correspond to the smaller surface-bonded sensors, group 3 includes PZT6 and PZT9, and, finally, group 4 includes sensors PZT7 and PZT8. These last two groups include sensors of the same type (P-876.SP1) and symmetric location.

Normal distribution of the four groups was checked with the Lilliefors-corrected Kolmogorov-Smirnov test. This test was used instead of Shapiro–Wilk test because this last became enough conservative when applied in this case. Considering that the normality of residuals assumption is the one that is least important for LMM analysis, since these models are robust even in the absence of normality in the data, Kolmogorov test is considered as valid. Table 3 shows the *p*-value of the residuals for the four groups of sensors as well as for all sensors considered jointly.

**Figure 9:**
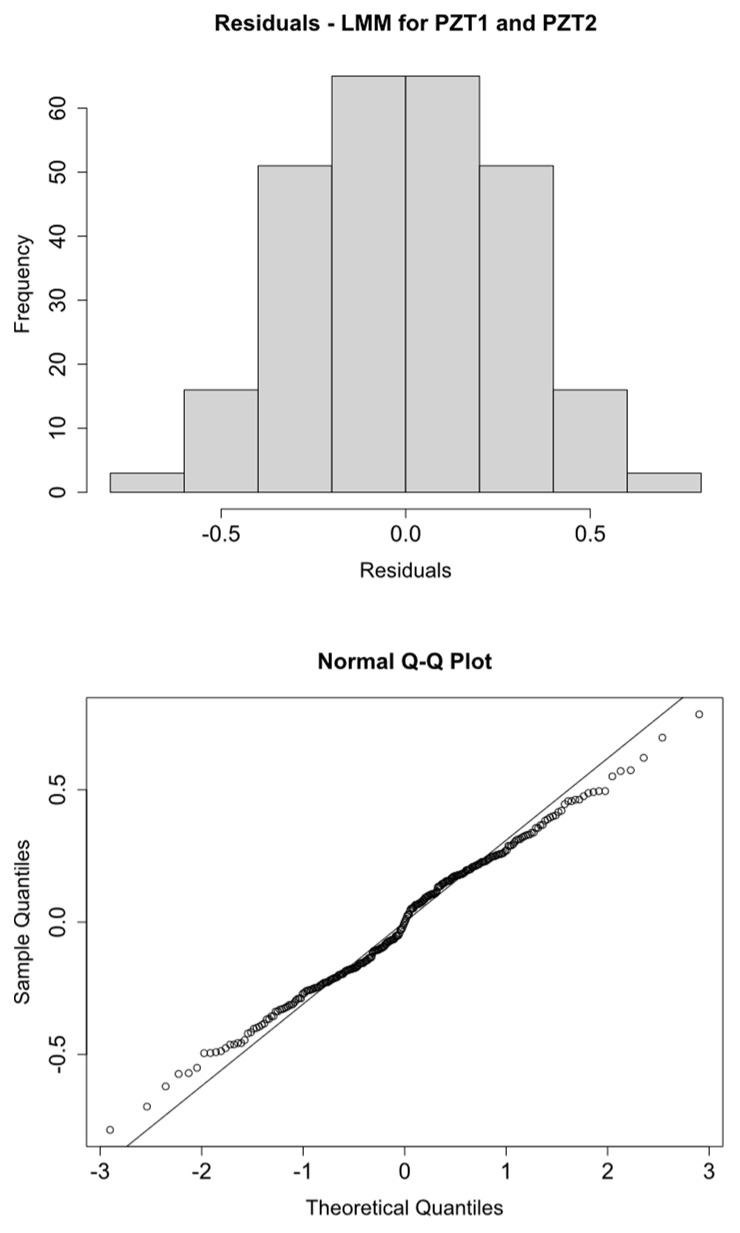
logRMSD histogram and Q-Q plot—Group 1.

**Figure 10:**
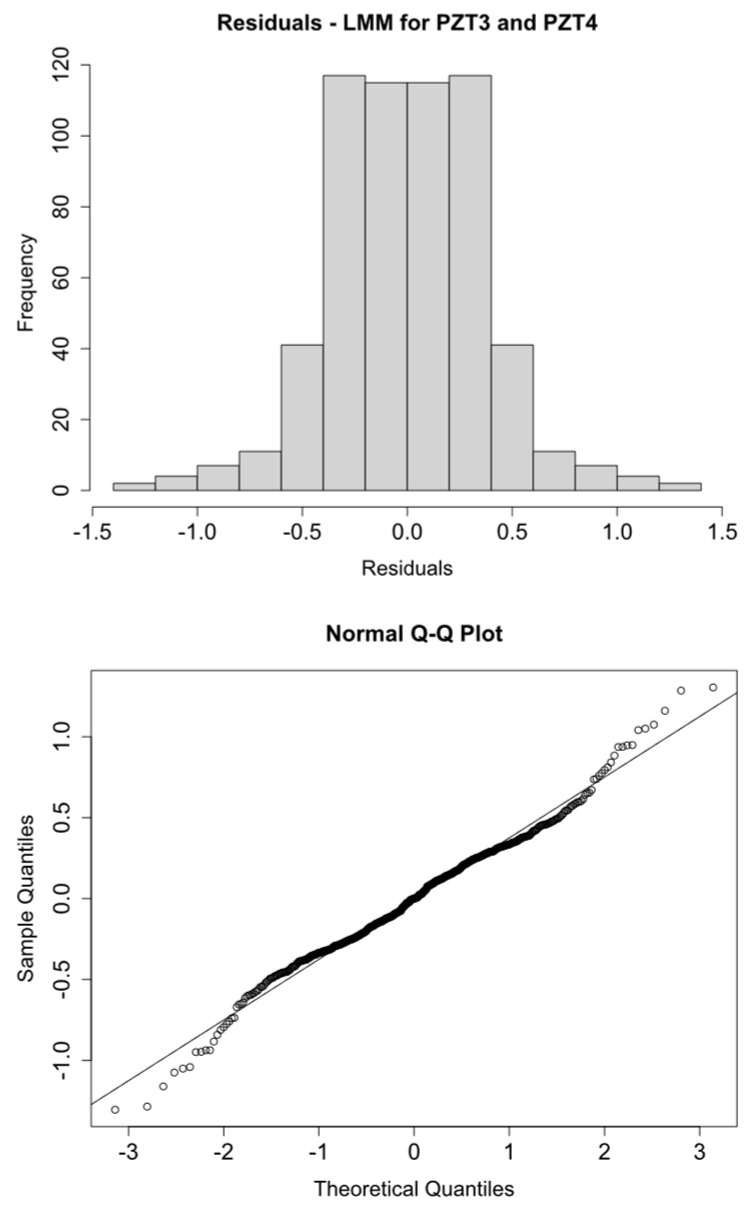
logRMSD histogram and Q-Q plot—Group 2.

**Figure 11:**
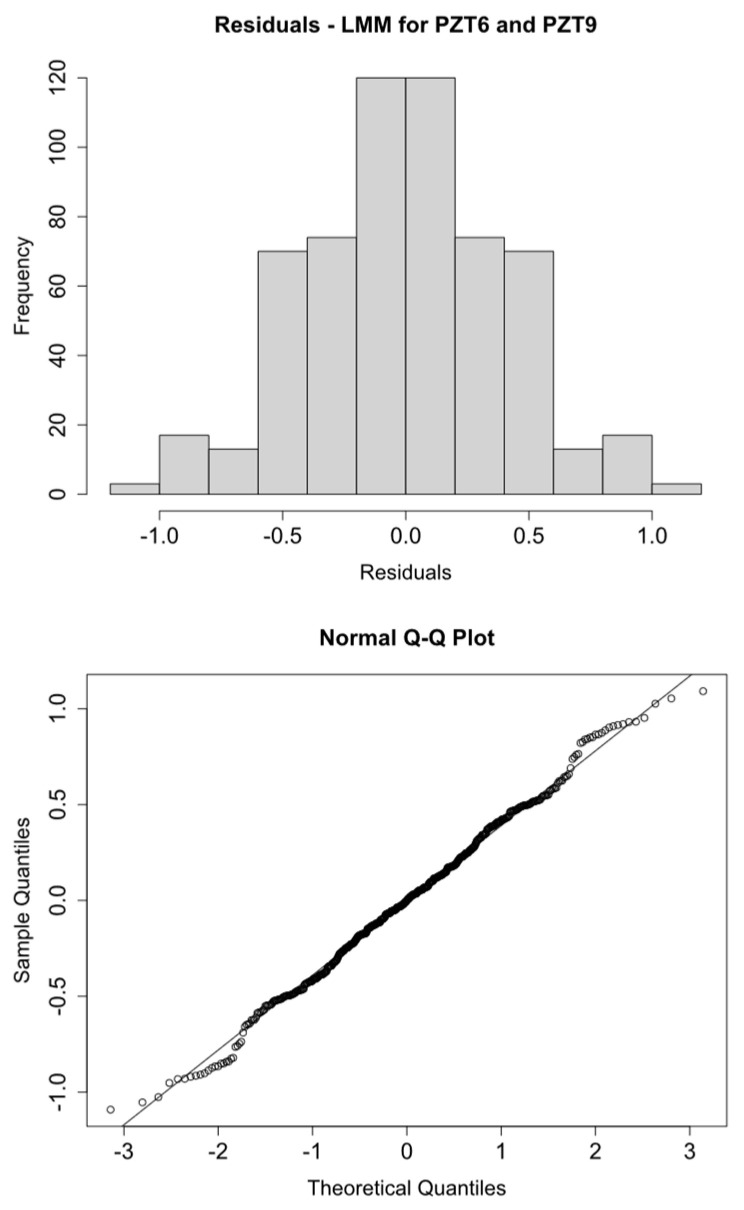
logRMSD histogram and Q-Q plot—Group 3.

**Figure 12:**
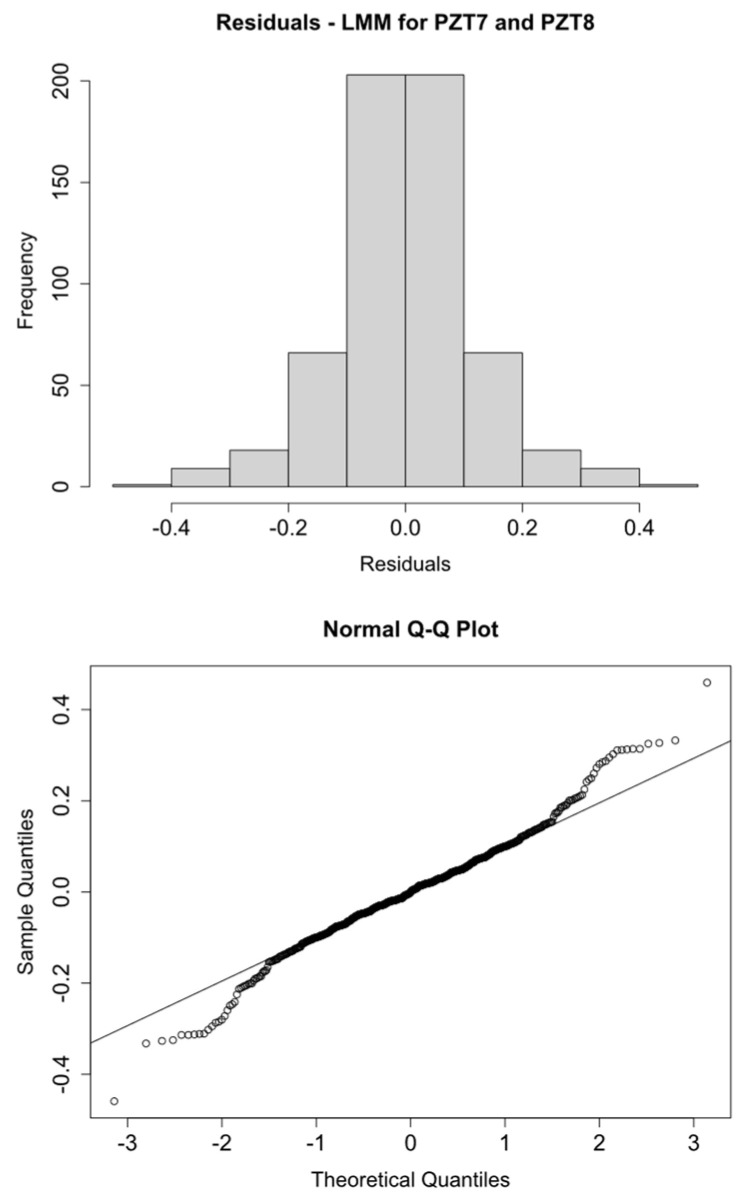
logRMSD histogram and Q-Q plot—Group 4.

Histograms and Q-Q plots confirm these results (Figure 9, Figure 10, Figure 11 and Figure 12).

**Figure 13:**
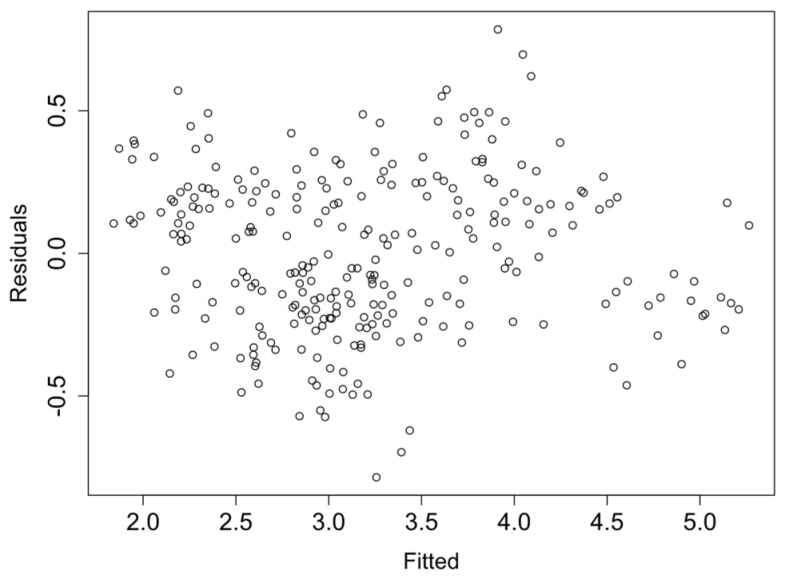
Fitted vs. residual values—Group 1.

**Figure 14:**
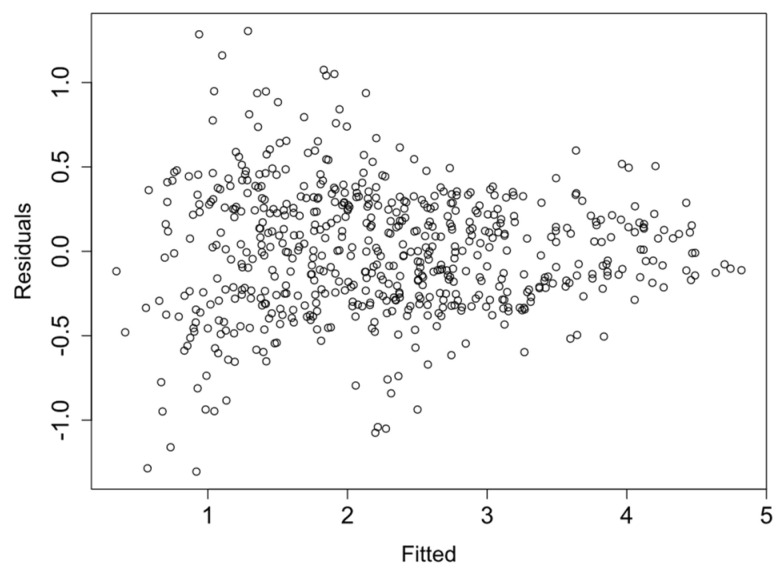
Fitted vs. residual values—Group 2.

**Figure 15:**
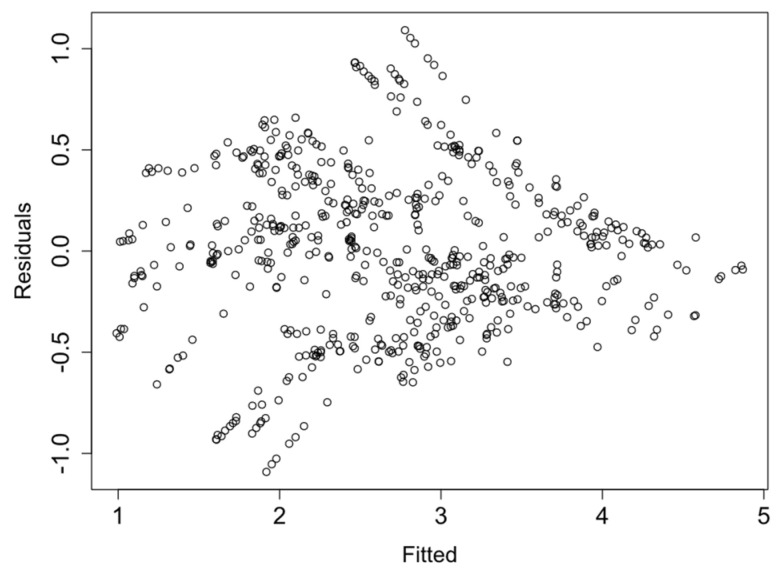
Fitted vs. residual values—Group 3.

**Figure 16:**
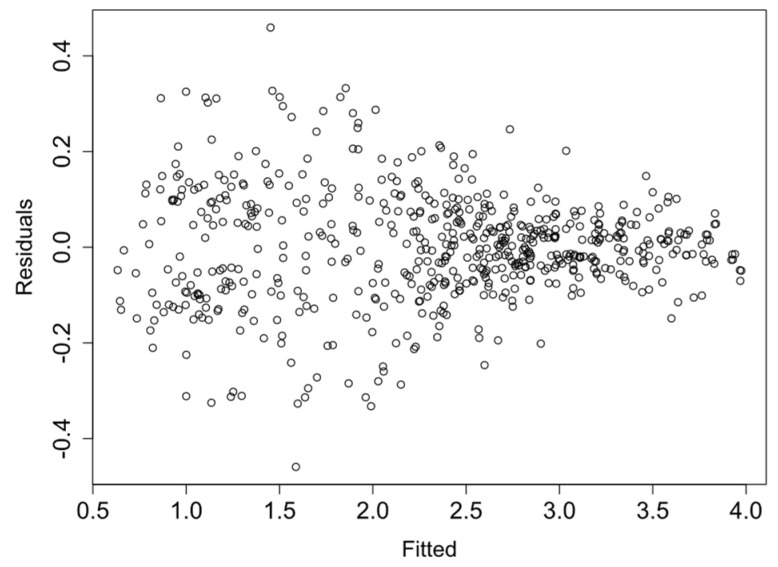
Fitted vs residual values—Group 4.

Finally, the linearity and homocedasticity of the logRMSD function are checked such as required by the model. Figure 13, Figure 14, Figure 15 and Figure 16 show the plots of the fitted values against the residuals of the model for each group of sensors. As the variance of the residuals is not dependent on the fitted value, the homocedasticity condition is not violated. Furthermore, none obvious pattern can be inferred in the residuals, and therefore the linearity assumption is also verified.

### 4.3. Statistical Analysis

All statistical calculations were carried out using the statistical software “R” version 1.3.1093 [26]. Mixed linear models have been adjusted using the library “lme4” [27].

LMMs enable to estimate fixed effects while properly taking into account the random variance associated with different participants, in this case the different sensors and experimental tests.

**Figure 17:**
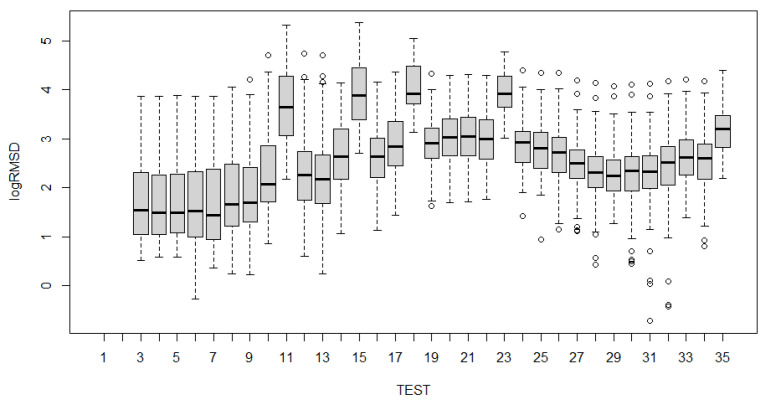
Boxplot for all sensors.

**Figure 18:**
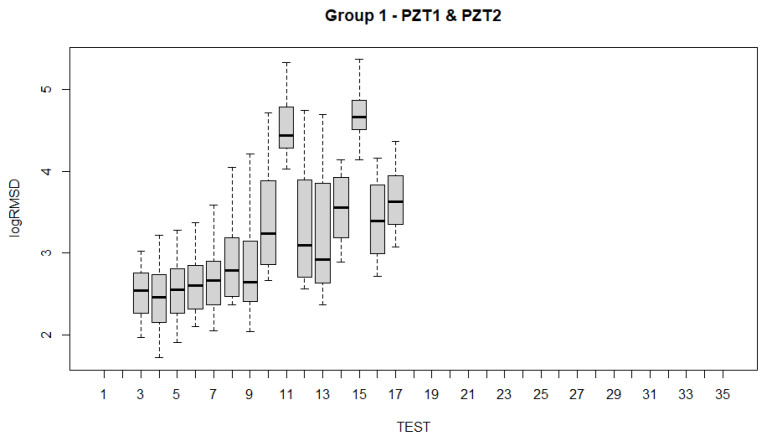
Boxplot for sensors of group 1.

**Figure 19:**
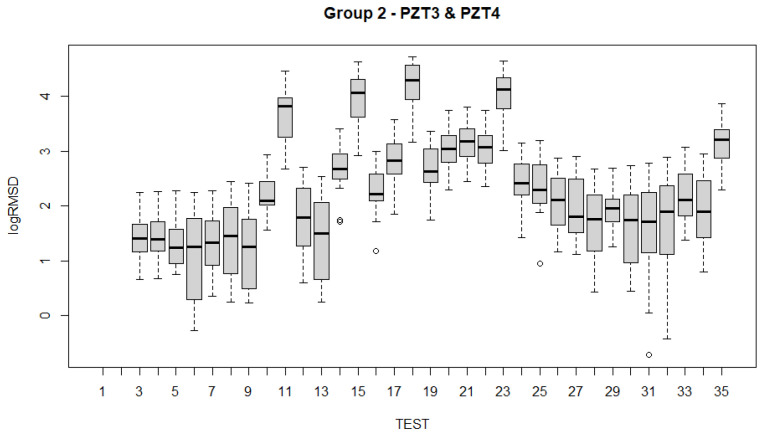
Boxplot for sensors of group 2.

**Figure 20:**
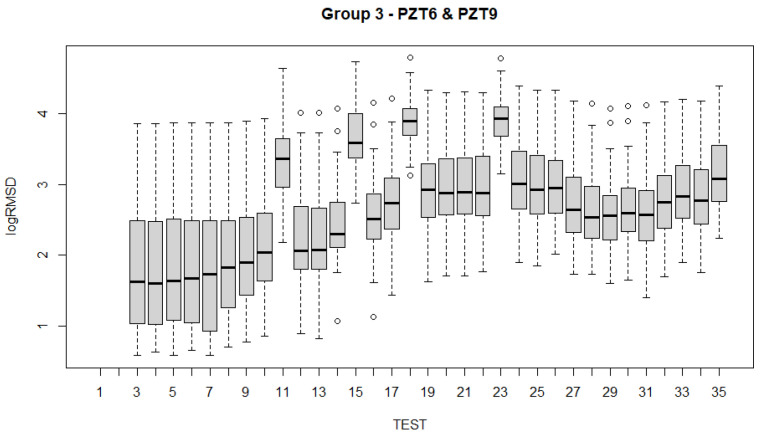
Boxplot for sensors of group 3.

**Figure 21:**
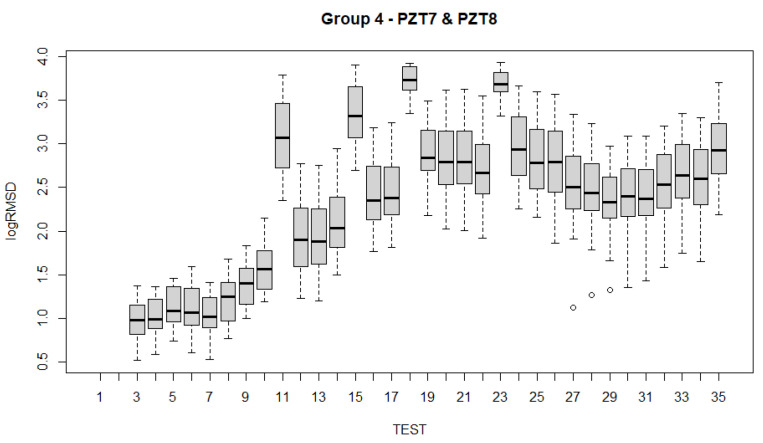
Boxplot for sensors of group 4.

To analyze the evolution of the variations of the fixed effects experienced by the specimen across the 35 experimental tests (Table 1) with the fitted LMMs, several alternatives are possible. Such examination can be carried out using test statistics and *p*-values. Boxplots have been constructed for all sensors and for each of the groups of sensors (Figure 17, Figure 18, Figure 19, Figure 20 and Figure 21). For group 1, only results until test 17 are shown since this group includes PZT2 sensor which came unglued partially during this test. Table 4 shows the *p*-values computed from the analysis of deviance for the LMM. A low *p*-value, such as observed in Table 4, means that the variations experienced by the specimen, both due to mechanical damage and temperature, affect significantly to the RMSD index. This confirms the suitability of this metric to assess the structural condition of the beam. In a further study, the frequency was also included as a fixed effect in the model and *p*-value was computed such as shown in the last row of Table 4. In this case, it is clear that the frequency does not have effects on RMSD coefficient.

**Figure 22:**
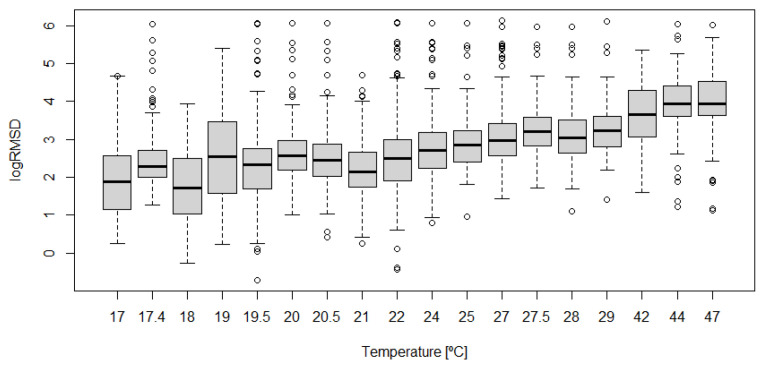
Effect of temperature on log(RMSD).

Boxplots shown in Figure 17, Figure 18, Figure 19, Figure 20 and Figure 21 show clearly how the increase of temperature (tests 11, 15, 18, and 23) affects in a higher proportion to RMSD than the mechanical variations introduced by the applied loads. This different sensitivity can be used to discriminate those variations due to the temperature from those due to mechanical damage. This conclusion is extensible to all groups of sensors. Boxplot of RMSD for the different temperatures reached along the test campaign has been also computed (Figure 22). It shows clearly the influence of the temperature on RMSD index and the sensitivity for each temperature value.

Now, if we compare the evolution of boxplots in Figure 17, Figure 18, Figure 19, Figure 20 and Figure 21 with the evolution of temperature of the specimen (red line in Figure 3), we observe clearly that the evolution is very similar which demonstrates the high sensitivity of the impedance to the temperature and can explain some of the phenomena occurring in the specimen. Any decrease of temperature is reflected by a decrease of log(RMSD), while any increase of temperature involves an increase of log(RMSD). This is clearly identified for tests 26 to 30 under 13.7 kN where an initial decrease of temperature is continued with a subsequent increase of temperature (Figure 3). The keypoint is how to filter this from the variations due to mechanical damage. For it, the identification of the results of Figure 18, Figure 19, Figure 20 and Figure 21 with Figure 3 and Figure 22 and the data contained in Table 1 helps to filter the observations. For instance, the results show that under sustained loads, except in case of a remarkable mechanical change, little variations are identified in log(RMSD) and those are probably due more to environmental temperature variations than to damage. In this sense, if we observe Figure 21 for tests 3 to 10 in comparison with Figure 18, Figure 19 and Figure 20, it is clear that some mechanical damage occurred near the sensors belonging to group 4. Additionally, some cracks developed around the external sensors during test 10. By using the same philosophy, it is clear that for the last test a severe damage occurred near all sensors with special focus on those sensors closer to the midspan (groups 2 and 3).

Another interesting point to remark which may affect to the interpretation of the results is that after any sustained loading test, some remanent strains stay in the specimen, especially in those more loaded areas. However, if we observe Figure 20 and Figure 21, corresponding to groups 3 and 4 of sensors, respectively, bonded directly to FRP, we can check too that after heating, when the beam recovers the environmental temperature, the remanent strain previous to the heating has grown. This phenomenon is much more remarkable for the sensors bonded to the most loaded zones (Group 4). To our knowledge, this observed behavior is due to the combined effect of the load removal, to the temperature increment provided by the heating, which originates an increase of tensile stresses in FRP because of its small thermal dilatation coefficient in comparison with steel and concrete, and to the delayed effects induced by the creep of the concrete. A temperature increment would induce an elongation in all materials. Considering that these elongations are not uniform, a small curvature would appear in the specimen, resulting in an increment of the tensile stress in the FRP with respect to the previous residual stress and an opening of the cracks. The delayed creep effects once the cooling has concluded may explain the presence of this remanent strain.

The high variance of the data observed in some of the boxplots makes difficult its interpretation and advises to carry out a further complementary pairwise analysis between the different tests to identify which pairs of tests differ significantly from each other. For simplicity, considering the high number of tests of the experimental programme, we will limit the pairwise study only to consecutive tests (Table 5). As in previous analyzes, a *p*-value smaller than 0.05 means that there is a significant difference in RMSD index between that pair of consecutive tests, and therefore the model has successfully distinguished them with a significant confidence.

From Table 5, the following conclusions can be extracted in combination with the boxplots:

(a) In general, the observed pattern for all groups of sensors according to the pairwise analysis across the tests is very similar in agreement with the boxplots, except for some differences which will be commented next;
(b) It is clear that the highest contrast in the *p*-values appears when heating of the specimen is performed. That high contrast is also shown when the specimen returns to the environmental temperature once its heating is interrupted. Therefore, high temperature variations are perfectly identified with *p*-values.
(c) For the first tests which were performed under a sustained load of 8 kN (tests 3 to 10), there is not a significant difference between consecutive tests except for test 10 when sensors of groups 1, 2 and 4 are considered. This test was the longest test of all those tests carried out under 8 kN and this same conclusion was derived from the boxplots.
(d) When a new sustained load test of 8 kN is performed after the heating and subsequent cooling of the beam, the groups of sensors fail to detect a significant difference in RMSD coefficient, except for group 2 between 13 and 14.
(e) For the sustained load tests under 9.3 kN, no significant difference was detected. The same occurs for the sustained tests under 13.7 kN. In this case, only a clear variation is identified for those sensors bonded to FRP close to the midspan when the beam is initially loaded up to 13.7 kN.
(f) The last load increment until reaching 19.6 kN shows a clear deterioration of the specimen clearly identified by the internal sensors. The much lower *p*-value in comparison with other previous values, except those due to heating/cooling may be a symptom of severe damage in the structure as the experimental tests demonstrated.

## 5. Discussion and Conclusions

The available research about the behavior of concrete beams strengthened with NSM-FRP systems when subjected to different levels of sustained loads and at ambient and elevated temperature is very limited or practically inexistent. However, its prediction is essential taking into account the sudden and brittle nature of the most common failure modes for this type of strengthening. Because of it, EMI technique based on high frequency impedance spectra has been used. This technique is also particularly sensitive to temperature variations. To give a suitable interpretation of the experimental impedance measurements, strain and temperature FBG sensors together with LMM analysis model have been used.

For the study, an extensive experimental programme has been carried out during more than 1.5 years on a concrete beam strengthened with a NSM-FRP laminate. In the work, three main difficulties have been addressed. On the one hand, the application of high levels of load which will introduce mechanical damage into the structure. On the other hand, the application of a load sustained along the time and the possible induced creep strains; and, finally, the consideration of elevated temperature variations which will influence on the performance of the strengthened beam. The complexity of these three aspects together with the hierarchical structure of the monitored data along the experimental programme using PZT based EMI method in different regions require of some methodologies able to address them suitably.

Linear mixed models provide an appropriate framework for the analysis of data which are likely to be correlated across the variability of an experimental programme as those derived from the monitoring of a structure along time and under different conditions. By using a linear mixed model, accounting for repeated measures among PZT sensors, as well as sensor level variance, it was found how the mechanical and thermal effects affected on RMSD metrics. Variations of temperature experienced by the beam are filtered from those variations due to mechanical damage. Additionally, the mechanical performance of the beam under sustained load is also collected by LMMs.

LMMs are becoming increasingly popular as a data analysis method in other scientific areas and researchers are encouraged to apply them rather than other analysis tools for structural monitoring purposes since they allow for the consideration of different phenomena in a direct and joint way giving a global vision about the structural performance but considering simultaneously the individuality of the sensors as well as of the different monitored stages.

Usually, in the proposed impedance-based methods to separate temperature variations from structural damage, temperature compensation techniques based on the use as a pattern of impedance measurements obtained from the structure in various thermal levels are applied. These techniques are applicable in a lab framework but, however, its application on real structures is more limited because of the lack of a reference pattern to do the compensation. In the proposed method the analysis is performed in a direct way using as a support FBG measurements and LLMs. Although this methodology is mainly qualitative, *p*-values derived from a pairwise analysis can be assumed as quantitative indicators of the severity of the experienced changes.

The lack of published works about this topic in the literature make difficult its comparison with other proposed approaches. Because of it, one of the topics to be covered in the near future should be the comparison of the proposed procedure with machine learning approaches since these methods are able to detect hidden patterns from monitored data. In the same way, another possible topic to be addressed in the far future is about the study of the performance of this type of structures under the effect of high loading rates, which is a still hardly explored topic.

## Figures and Tables

**Table 1:** Loading history.

Test Number	dd/mm/yyyy	Sustained Load Level [kN]	Loading Test Duration [days]	Test Temperature [°C]	
0	08/01/2018	0	-	NA	NA
1	11/01/2018	8	2	17	Environmental
2	25/01/2018	8	7	19	Environmental
3	08/02/2018	8	14	17	Environmental
4	13/02/2018	8	4	17	Environmental
5	15/02/2018	8	1.5	17	Environmental
6	19/02/2018	8	3	18	Environmental
7	22/02/2018	0	3	18	Environmental
8	12/03/2018	8	14	19.5	Environmental
9	03/04/2018	8	21	19	Environmental
10	24/04/2018	8	21	22	Environmental
11	26/04/2018	0	1	42	Heated
12	30/04/2018	0	3	22	Environmental
13	03/05/2018	0	3	21	Environmental
14	27/05/2018	8	23	24	Environmental
15	31/05/2018	0	3	42	Heated
16	01/06/2018	0	2	24	Environmental
17	02/07/2018	8	30	27	Environmental
18	05/07/2018	0	3	47	Heated
19	09/07/2018	0	3	27	Environmental
20	25/07/2018	8	14	28	Environmental
21	26/07/2018	9.3	1	27.5	Environmental
22	04/09/2018	9.3	31	27	Environmental
23	05/09/2018	0	1	44	Heated
24	07/09/2018	0	3	27	Environmental
25	06/10/2018	9.3	21	25	Environmental
26	07/10/2018	13.7	1	22	Environmental
27	06/11/2018	13.7	28	20	Environmental
28	04/12/2018	13.7	29	20.5	Environmental
29	04/02/2019	13.7	60	17.4	Environmental
30	13/03/2019	13.7	41	19.5	Environmental
31	14/03/2019	17.7	1	19.5	Environmental
32	13/05/2019	17.7	60	22	Environmental
33	10/06/2019	17.7	30	24	Environmental
34	13/06/2019	19.6	2	24	Environmental
35	27/07/2019	19.6	42	29	Environmental

**Table 2:** Kolmogorov-Smirnov test.

**Test number**	**3**	**4**	**5**	**6**	**7**	**8**	**9**
*p*-value	0.0676121	0.17934489	0.07940822	0.46347637	0.10371856	0.15253014	0.28992103
**Test number**	**10**	**11**	**12**	**13**	**14**	**15**	**16**
*p*-value	0.0568042	0.81895703	0.60769883	0.49746651	0.91459634	0.79595994	0.69038784
**Test number**	**17**	**18**	**19**	**20**	**21**	**22**	**23**
*p*-value	0.86443078	0.36518012	0.9042454	0.89197109	0.96226618	0.90848409	0.91443105
Test number	24	25	26	27	28	29	30
*p*-value	0.85373292	0.95754631	0.70759643	0.5601079	0.43779154	0.73708194	0.49989917
**Test number**	**31**	**32**	**33**	**34**	**35**		
*p*-value	0.05344414	0.04345163	0.84320087	0.33456275	0.95263826		

**Table 3:** Kolmogorov test—Groups of sensors.

Group Number	*p*-Value
All sensors	0.05832
1	0.04118
2	0.06561
3	0.7728
4	0.0085

**Table 4:** Analysis of deviance—*p*-value.

	All Sensors	Group 1	Group 2	Group 3	Group 4
Variations	<2.2 × 10^−16^	2.0 × 10^−16^	2.0 × 10^−16^	2.0 × 10^−16^	2.0 × 10^−16^
Frequency	1	0.99999	0.99879	1	2.0 × 10^−16^

**Table 5:** Pairwise analysis between consecutive tests—*p*-value.

Tests	*p*-Value				
	All Sensors	Group 1	Group 2	Group 3	Group 4
3–4	1	1	1	1	1
4–5	1	1	1	1	1
5–6	1	1	1	1	1
6–7	1	1	1	1	1
7–8	1	1	1	1	0.013256
8–9	1	1	1	1	0.459903
9–10	0.00000189	0.000224	0.000013	1	0.036852
10–11	<2 × 10^−16^	8.67 × 10^−12^	7.11 × 10^−13^	0.00000351	2 × 10^−16^
11–12	<2 × 10^−16^	2.26 × 10^−13^	2 × 10^−16^	0.0000437	2 × 10^−16^
12–13	1	1	1	1	1
13–14	0.00000716	0.778527	1.52 × 10^−8^	1	0.09984
14–15	<2 × 10^−16^	4.42 × 10^−13^	7.57 × 10^−10^	0.00000138	2 × 10^−16^
15–16	<2 × 10^−16^	2 × 10^−16^	2 × 10^−16^	0.0000519	2 × 10^−16^
16–17	0.280975	0.977147	0.449131	1	1
17–18	<2 × 10^−16^		3.35 × 10^−11^	0.00000479	2 × 10^−16^
18–19	<2 × 10^−16^		9.06 × 10^−14^	0.000369	2 × 10^−16^
19–20	1		1	1	1
20–21	1		1	1	1
21–22	1		1	1	1
22–23	<2 × 10^−16^		0.0000125	0.000254	2 × 10^−16^
23–24	<2 × 10^−16^		2 × 10^−16^	0.003112	2 × 10^−16^
24–25	1		1	1	0.541478
25–26	1		1	1	1
26–27	1		1	1	0.0000966
27–28	1		1	1	1
28–29	1		1	1	1
29–30	1		1	1	1
30–31	1		1	1	1
31–32	1		1	1	0.569178
32–33	0.494927		0.263567	1	1
33–34	1		1	1	1
34–35	6.22 × 10^−11^		4.85 × 10^−9^	1	3.8 × 10^−8^

## Data Availability

The data presented in this study are available on request from the corresponding author. The data are not publicly available due to privacy reasons.
